# The Impact of Selected Physical Factors on Neurogenesis—What Do We Know?

**DOI:** 10.3390/cells15211917

**Published:** 2026-10-22

**Authors:** Adam Lipski, Kamil Koszela, Julia Pingot, Michał Słupiński, Robert Irzmański, Marta Woldańska-Okońska

**Affiliations:** 1 Department of Internal Medicine, Rehabilitation and Physical Medicine, Medical University of Lodz, 90-647 Lodz, Poland; 2 Department of Musculoskeletal Disorders, National Institute of Geriatrics, Rheumatology and Rehabilitation, 02-637 Warsaw, Poland; 3 Department of Nursing, Piotrkowska Academy, 97-300 Piotrków Trybunalski, Poland; 4 FiortClinic—Physiotherapy, Orthopedics and Rehabilitation, 97-300 Piotrków Trybunalski, Poland

**Keywords:** neurogenesis, physical factors, markers

## Abstract

**Highlights:**

**What are the main findings?**

• The physical factors listed in the study mostly have a positive effect on neurogenesis. Some of the physical factors studied, when appropriately selected, can be effectively used in the treatment of neurodegenerative diseases, central nervous system injuries, and in adaptation to specific conditions, or to improve cognitive processes in relation to the regulation of neurogenesis.

**What are the implications of the main findings?**

• A thorough understanding of physical conditions can protect against the adverse effects of certain physical phenomena or their specific parameters on the conditions for the proliferation and maturation of nerve cells—an issue that has not yet been sufficiently addressed in therapy and real life.

**Abstract:**

New discoveries are constantly being made regarding cognitive processes, pain perception, the development of degenerative diseases, brain aging, and many other functions of the nervous system. Neurogenesis plays a significant role in all of these processes. As a manifestation of brain plasticity, neurogenesis continues throughout life, and its intensity can vary depending on internal and external factors. The process by which neurons are generated can be disrupted. This depends, among other things, on age and represents one of the potential targets for interventions aimed at improving cognitive function. Inhibition of neurogenesis increases the risk of neurodegenerative diseases, including Alzheimer’s disease, Parkinson’s disease, and other conditions that lead to dependence on others in their advanced stages. The status of the neurogenesis process can be monitored by measuring the levels of mediators in the blood. The state of the brain can be influenced by providing stimuli that increase the concentration of mediators stimulating neurogenesis. People are exposed to physical factors that can inhibit the neurogenesis process, but there are also phenomena that stimulate it. Magnetic stimulation, ultrasound, electrostimulation, light therapy, normobaric and hyperbaric oxygen therapy, whole-body cryotherapy, as well as touch and vibrations, can have a significant impact on neurogenesis and accelerate this process. The aim of this study is to present the impact of physical factors on the process of neurogenesis in humans and animals, which can significantly influence the course of this process. The review uses the wording originally used in the source articles.

## 1. Introduction

The abilities of the human brain that are unique to humans include the ability to think abstractly, speak, write, and create objects or works of art, such as music. Generally, the mammalian cerebral cortex represents the highest point of brain development, reaching maximum complexity in terms of the number of neurons, their diversity, and functional circuits. The emergence of this unique complexity begins during embryonic development, when a limited number of neural stem and progenitor cells are capable of generating countless neurons in the appropriate numbers, types, and proportions through a process called neurogenesis. This process involves glial cells, synaptic connections, and the extracellular space. The evolutionary processes according to Villalba A. [1] that led to the highest degree of brain complexity are caused by neurogenesis, i.e., the formation of new nerve cells [1], in which they participate. Most of the studies cited below were conducted on animals as experimental physioanatomical investigations. In contrast, studies on humans consisted mainly of testing markers and examining markers, as well as postmortem observations.

The phenomenon of neurogenesis occurs throughout life; it is most dynamic at the beginning of human life, and with age, the formation of new neurons slows down, but it does not cease entirely [2,3]. There are many areas in the mature brain where new neurons are formed. Knowledge of the regulation of cortical neurogenesis—ranging from the description of progenitor cell types and their lineage relationships to the factors determining neuronal fate, as well as molecular, genetic, and epigenetic regulatory mechanisms—describes the evolution of cortical neurogenesis in vertebrates and leads to an understanding of human development [1].

In mammals, neural stem cells (NSCs) are found in the neocortex, striatum, substantia nigra, amygdala, hypothalamus, and along the third and fourth ventricles; however, these regions exhibit very low proliferative potential [4,5,6]. In the adult brain, two active zones containing NSCs are distinguished: the subventricular zone (SVZ) of the lateral ventricles and the subgranular zone (SGZ) of the hippocampal dentate gyrus. The SVZ (subventricular zone) contains the largest number of active cells, making it the primary source of new neurons in the adult animal brain. They are located beneath the lining of the lateral ventricles [4,5,6].

### Factors That Stimulate and Inhibit Neurogenesis

Neurogenesis in adults is, among other things, an important component of neuronal plasticity, the maintenance of brain homeostasis, and the preservation and remodeling of tissues in the central nervous system [7]. In a groundbreaking study conducted by Eriksson et al. [8], BrdU (5-bromo-2′-deoxyuridine)—a synthetic thymidine analog that is incorporated into the DNA of dividing cells—was used for immunofluorescent labeling, along with one of the following neuronal markers: calbindin, NeuN, or neuron-specific enolase (NSE). It was demonstrated that neural stem cells, tagged with these markers, arise after dividing progenitor cells in the dentate gyrus of adult humans. These results also suggest that the human hippocampus continues to generate new neurons throughout life [8]. This study marked a breakthrough in the world of science, proving that Santiago Ramón y Cajal’s nearly century-old theory regarding the lack of plasticity in the nervous system—according to which cells could ultimately die as a result of injury, degeneration, or development, but could not undergo changes or multiply—is, in fact, incorrect [9].

Both internal and external factors determine the migration and proliferation of neural stem cells (NSCs) in critical regions of the brain. The differentiation of stem cells into neurons or glial cells is influenced by neurotrophins, antidepressants, opioids, physical activity, glucocorticoids, sex hormones, growth factors, and activation of the N-methyl-D-aspartate (NMDA), which, through a common pathway, regulate neurogenesis in the dentate gyrus and hypothalamus, influencing learning processes, physical activity, stress, and diet [7], as demonstrated in experiments on mice.

Factors that stimulate neurogenesis include pharmacological interventions or physical treatments that increase serotonin levels. Properly high serotonin levels create favorable conditions for the formation of new neurons (for example, taking SSRIs—selective serotonin reuptake inhibitors). However, its mechanism of action is complex and depends on the receptor, the duration of exposure, and the conditions [7,10]. One factor that inhibits the neurogenesis process is, among other things, elevated stress levels due to the effects of cortisol, or depression caused by low serotonin levels. [7,10,11,12]. Adult hippocampal neurogenesis is important for the brain’s plasticity, including cognitive functions, learning, pattern recognition, and spatial memory. Furthermore, it is significant for certain emotional behaviors, such as depression and anxiety. However, the mechanisms related to neurogenesis in the hippocampus of healthy adults as their brains age require further research [12]. Neurogenesis in the hippocampus of adults is also influenced by acetylcholine and dopamine [9]. While stress significantly decreases neurogenesis through the influence of cortisol, the effects of acetylcholine (ACh) and dopamine (DA) depend on the type of receptors that transfer their signals, resulting in different outcomes [13]. A more detailed overview of these phenomena is beyond the scope of this review.

Neurogenesis in the adult mammalian brain is regulated by thyroid hormones and shows both specific and overlapping effects in different neurogenic niches [13]. Thyroid hormone regulates the survival of progenitor cells in the hippocampal SGZ and the differentiation of neuronal cells. It also influences the differentiation of progenitor cells in the SVZ, the completion of the cell cycle, and the determining of the future destination of neuronal cells [13].

The fact that neurogenesis in adults with neurodegenerative diseases is impaired may suggest that the regenerative ability of stem cells in the adult human brain is reduced or may be lost [12], which decreases the number of existing neurons. It is worth noting that neurogenesis can be influenced by internal factors, or by external factors, if signaling pathways are regulated by components of the extracellular environment, for example diet [12]. Specific changes in the gut microbiome have been observed in patients with Alzheimer’s disease (ALZ) and in rodent models. More importantly, recent studies suggest that the symptoms of ALZ may be transmitted to a healthy, young organism via the gut microbiota, which confirms its key role in Alzheimer’s disease [14]. Furthermore, adult hippocampal neurogenesis is considered a key cellular mechanism responsible for cognitive changes influenced by both systemic and intestinal factors in ALZ [14]. This discovery is of significant importance for new treatments for this disease, especially since, according to the WHO, there were approximately 57 million people living with dementia worldwide in 2021 (the majority of whom had ALZ), and nearly 10 million new cases are added each year [15].

Internal factors influencing neurogenesis include, for example, age, hormones, neurotrophic factors, and inflammatory mediators such as interleukin 1β (IL-1β), which inhibit various phases of neurogenesis, such as proliferation and maturation. External factors regulating neurogenesis in the hippocampus in adults include pathological stimuli such as pain and hypoxia [12,16,17]. This process may also be influenced by external factors such as alcohol, radiation exposure, and malnutrition. Alcohol disrupts neurogenesis in adults by suppressing BDNF expression in the hippocampus; however, this process may be reversible upon discontinuation of alcohol intake [12,16,17].

In addition to the typical symptoms associated with the COVID-19 pandemic, infection with this virus is also linked to the development of inflammatory and degenerative diseases of the nervous system. The specific mechanisms underlying neurogenesis disorders and the neurological deficits caused by this infection remain largely unknown. There appears to be a number of evidence suggesting that SARS-CoV-2 infection affects neurogenesis negatively and is also linked to accelerated neuronal cell death [18].

Jiang M. et al. [12] describe external factors associated with neurogenesis disorders. Among the most significant external factors supporting this process, physical factors play a key role. These include factors such as temperature, light, magnetic fields, and mechanical stimuli (e.g., physical exercise) [19]. Factors that inhibit neurogenesis include ionizing radiation used, for example, in radiation therapy [20]. Physical factors, on the other hand, may stimulate neurogenesis through pathways that increase levels of brain-derived neurotrophic factor (BDNF), improving cerebral blood flow, reducing oxidative stress and inflammation, modulating the HPA axis (hypothalamic-pituitary-adrenal axis), or stimulating neuroplasticity [21]. These factors will be discussed in greater detail in the following subsections.

The development of the central nervous system (CNS) is a dynamic process affected by environmental, genetic, biochemical, and physical factors, beginning in the early stages of embryonic development and continuing until maturity is reached. Key regulatory processes include transcriptional, post-transcriptional, and epigenetic factors, as well as countless signaling pathways which influence the fate of neural progenitor cells and newly formed neurons that are capable of migration. Epigenetic factors play a variable but very important role, as they can serve as regulatory elements originating from outside the organism that we can influence [21].

Impairment of the regulatory system, caused by mutations in its fundamental components, can lead to developmental abnormalities of the cerebral cortex, epilepsy, intellectual disability, and autism in humans. Understanding the normal development of the human central nervous system (CNS) is therefore crucial for understanding the mechanisms responsible for neurodevelopmental disorders and, consequently, for developing effective and targeted therapeutic strategies [2,6,12,21].

**Figure 1:**
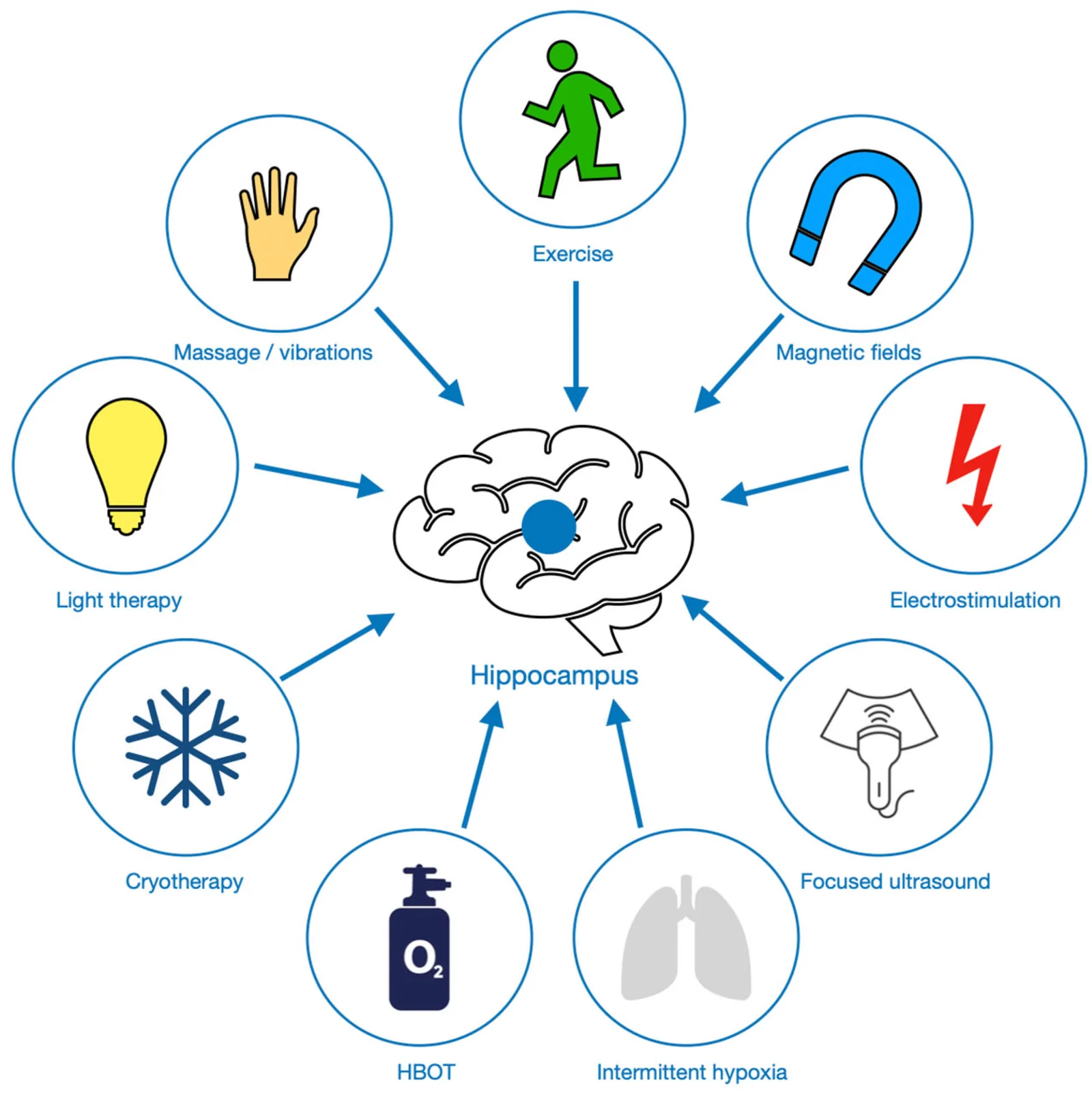
The physical factors that stimulate neurogenesis.

External and internal factors can have either a stimulating or a negative effect on neurogenesis. Understanding these mechanisms may reveal opportunities to optimize the neurogenesis process and support regeneration. For example, this can be achieved through the use of specific medications, environmental enrichment, exposure to scents, aerobic exercise, or physical factors. This is precisely why this article focuses on the role of physical factors in neurogenesis, as in many cases they appear to have a positive effect on it (Figure 1). However, if it were to turn out that some of them are harmful, it would be advisable to issue warnings and avoid them in the human environment.

The objective of this research is to present the impact of physical factors on the process of neurogenesis in humans and animals, as they are more ubiquitous than ingested substances and can significantly influence the course of this process.

## 2. The Effect of Physical Factors on Neurogenesis

### 2.1. The Effect of Physical Exercise on Neurogenesis (Aerobic and Strength Training)

To the present, evidence regarding the role of physical activity in neurogenesis and cognitive functions has been based primarily on studies of aerobic exercise. In a study by Ben-Zeev T. et al. [22], three types of exercise— anaerobic, aerobic, and resistance—were described as having a beneficial effect on brain plasticity and cognitive functions. However, the mechanisms stimulating these changes differ across these types of training. Anaerobic and aerobic exercise affect the brain by altering the expression of BDNF, lactate, vascular endothelial growth factor (VEGF), and certain other proteins in the brain, including myokines, among others. In turn, resistance training appears to influence brain plasticity through myokines secreted by skeletal muscles, such as irisin, insulin-like growth factor 1 (IGF-1), and BDNF, which stimulate neurogenesis in the brain. In addition to various forms of training, modifications to the program—including intensity, load, and rest intervals between exercises—lead to numerous possible training regimens, which can stimulate neurogenesis in various ways [22,23].

Studies conducted on animals and humans have shown that physical exercise improves mental status and cognitive function. At the same time in the dentate gyrus, physical activity increases neurogenesis of the hippocampus, which is responsible for learning and memory. The positive correlation between running and neurogenesis suggests that new hippocampal neurons may mediate the improvement in learning associated with physical exercise [12,23].

Physical exercise influences the reversal of brain aging as a systemic intervention. It was revealed that in the administration of plasma-circulating factors caused by exercise, aged mice transferred the effects of exercise on neurogenesis and cognitive function to sedentary adult mice. It was found that plasma specific phospholipase D1 (Gpld1) concentrations—glycosylphosphatidylinositol (GPI), a liver-derived GPI-degrading enzyme, increased after exercise and correlated with improved cognitive function in elder mice. Furthermore, blood Gpld1 levels were elevated in active, healthy older adults. In this way, a liver-brain axis was identified through which blood factors may mediate the benefits of physical exercise in older adults [24].

### 2.2. The Effect of Magnetic Fields on Neurogenesis

Severe brain damage can be induced during radiation therapy for head and/or neck tumors (RIBI—radiation-induced brain injury), which is caused, among other things, by pyroptosis. Unlike apoptosis, pyroptosis releases the cell’s contents into the surrounding tissue, triggering severe inflammation [25]. During radiation therapy, this mechanism damages cancer cells while also causing damage to healthy tissues.

A noninvasive method of brain stimulation, repetitive transcranial magnetic stimulation (rTMS) is used in various neurological disorders; in particular, rTMS is effective in treating RIBI. A study by Qin T. et al. [26] demonstrated that rTMS significantly alleviates cognitive impairments caused by RIBI by reducing microglial pyroptosis and promoting hippocampal neurogenesis through the BDNF pathway.

Deep brain stimulation (DBS) is similar to magnetic therapy (bi-phasic waveform) in terms of waveform and frequency (1–20 Hz), but is characterized by significantly higher magnetic field intensities, ranging from 1.5 to 3.0 Tesla. DMS not only stimulates the proliferation of mature neural progenitor cells in DG but as well as promotes development and maturation of newly formed neurons, suggesting that these neurons are capable of integrating into the hippocampal neural circuits and contributing to neural plasticity of central nervous system [27].

DMS exhibits antidepressant effects in a mouse model of learned helplessness and restores long-term hippocampal plasticity in rats whose function had been impaired by immobilization-induced stress. Furthermore, DMS attenuates the mouse model of stress response in a Rett syndrome and as a result significantly prolongs the lifespan of these animals. Long-term treatment with rTMS has demonstrated marked antidepressant effects in animal models and may promote neurogenesis in adults under conditions of chronic stress. rTMS restores long-term potentiation (LTP) in the hippocampus of rats and function of which has been disrupted by immobilization-related stress [27]. Since studies indicate an interaction between glutamate and gamma-aminobutyric acid (GABA), where metabotropic glutamate receptors (mGluRs) can modulate GABA release, affecting the excitation-inhibition balance in the hippocampus, DMS may positively influence glutamate secretion or GABA inhibition. It is also possible that DMS affects serotonin, dopamine, norepinephrine, and acetylcholine, which act as neuromodulators, influencing synaptic plasticity, memory, and learning processes [27].

### 2.3. The Effect of Electrostimulation on Neurogenesis

Non-invasive brain stimulation (NIBS) techniques have gained importance as potential therapeutic tools aimed at improving the outcomes of cognitive rehabilitation. NIBS methods include transcranial electrical stimulation (tES) (including transcranial direct current stimulation (tDCS), and high-resolution tDCS, transcranial alternating current stimulation (tACS) as well as transcranial magnetic stimulation (TMS) (described above) and transcranial random noise stimulation (tRNS). Most of the studies analyzed demonstrated a positive effect of tDCS on the most neurocognitive disorders [28,29]. Brain stimulation as non-invasive, including repetitive transcranial magnetic stimulation (rTMS) and transcranial tDCS, utilizes neuroplasticity in the treatment of numerous disorders, like Parkinson’s disease, stroke, autism, and depression [30].

Repeated tDCS stimulation at a current density below the threshold for neuronal damage led to a polarity-dependent reduction in the baseline expression in the mouse cerebral cortex of ionized calcium-binding adapter molecule 1 (Iba1, known as a 17-kDa calcium-binding protein specifically expressed in macrophages and microglia). Since Iba1 is expressed only in the active state, rather than resting, microglial cells, both upregulation and downregulation of Iba1 present evidence for functional modification of microglial subpopulations by tDCS. Contrary, both anodal and, to a greater degree, cathodal tDCS induced neurogenesis in SVZ. These findings were described in terms of the size of the immunoreactive area. The data suggests that tDCS exerts its effects via multifaceted mechanisms, including neurogenesis and immunomodulation, which supports the use of tDCS to induce regeneration and facilitate functional recovery [31].

The study described in the article by Moyal M. et al. [32] involves the treatment of seventy participants with lorazepam-resistant catatonia, who may undergo 20 sessions of active or sham tDCS, administered twice a day with a 2 mA current intensity for 20 min, with anodal stimulation of the left dorsolateral prefrontal cortex (DLPFC) and cathodal stimulation of the left temporo-parietal junction (TPJ). In addition to daily clinical assessment, patients will undergo functional, structural and diffusion magnetic resonance imaging (MRI), an electroencephalogram (EEG) of64-channel resting-state, and blood tests for markers of inflammation, neurogenesis and genetic markers, before, during and after treatment. The hypothesis of an excitation/inhibition (E/I) imbalance of catatonia pathophysiology, supported by the effectiveness of GABA-A agonists [27], the brain anti-inflammatory factor NMDA and studies of neuroimaging, suggests that EEG markers reflecting neuronal E/I balance will help to the identification of diagnostic biomarkers and expand our insight of the pathophysiology of catatonia [32]. The hypothesis regarding an imbalance between excitation and inhibition (E/I) in the pathophysiology of catatonia—supported by the efficacy of GABA-A receptor agonists [27], the cerebral anti-inflammatory factor NMDA, and neuroimaging studies—suggests that EEG markers reflecting the E/I balance in neurons will aid in the identification of diagnostic biomarkers and expand our understanding of the pathophysiology of catatonia. This is of particular importance for understanding and potentially treating this complex syndrome of psychopathological symptoms.

### 2.4. The Effect of Ultrasounds on Neurogenesis

Growing evidence points to the possibilities of transcranial focused ultrasound to improve brain function and available repair. Low-intensity focused ultrasound (LIFU) allows for accurate, non-invasive modulation of neuronal activity, promoting cellular repair and plasticity of the brain [33]. By targeting specific areas of the brain, LIFU strengthens synaptic connectivity, facilitates the release of neurotrophic factors, and modulates the molecular and cellular pathways necessary for neuronal regeneration. These mechanisms work closely together to support proliferation, differentiation, and neural functional integration, leading to improved cognitive function and neuroprotection without causing thermal damage. A comprehensive review of the effects of LIFU [33] on brain function highlights its role in cellular adaptation, neuromodulation, and long-term utility in the treatment of neurodegenerative diseases and brain injuries. The review covers optimal ultrasound parameters, potential efficacy in cellular and behavioral models, and its therapeutic potential in functional restoration and brain repair. By expanding its scope beyond isolated mechanisms, LIFU is becoming a versatile neuromodulation tool with significant clinical potential for improving cognitive outcomes and brain resilience [33].

Ultrasound (US), particularly LIFU and transcranial ultrasound stimulation (TUS), have a stimulating effect on neurogenesis—the process of new neuron formation from stem/progenitor cells in the adult brain (first and foremost in the hippocampus and SVZ). The effects observed by studies (in animal models and in vitro) include increased proliferation of progenitor cells (BrdU+, Nestin+), increased differentiation into neurons (β-III-tubulin+, NeuN+), and increased migration of new neurons to the cortex and hippocampus [33].

LIFU may alleviate the motor symptoms of Parkinson’s disease by adjusting microglial activation, increasing levels of neurotrophic factors, limiting oxidative stress, and promoting regeneration and nerve repair. LIFU combined with microbubbles (MB) may facilitate drug penetration across the blood–brain barrier (BBB), which could become a new direction in the treatment of Parkinson’s disease. The observed neurotrophic factors include BDNF, GDNF (promoting the growth and differentiation of dopaminergic neurons), including NTN (Neurturin), and the paracrine fibroblast growth factor-20 (FGF20). Importantly, improvements in cognitive function (spatial memory, learning) were observed. Moreover, the observed effects are long-term (lasting up to several weeks after stimulation), indicating the occurrence of biological hysteresis due to the delayed effects of the physical stimulus [34]. LIPU promotes the expansion, differentiation, and migration of mesenchymal stem cells, and it has been reported that LIFU-induced BBB regulation can promote neurogenesis through the activation of endogenous neural stem cells. Low-intensity pulsed ultrasound LIPU similar to LIFU promotes the expansion, differentiation, and migration of mesenchymal stem cells, and it has been reported that LIFU-induced BBB regulation can promote neurogenesis through the activation of endogenous NSCs [33,34].

The effective parameters for activating neurocyte stem cells are reported as follows: frequency: 0.5–1.5 MHz, intensity: <500 mW/cm^2^ (below the thermal threshold), pulse duration: 0.1–1 ms, and frequency: 1–10 Hz. Exposure lasts 1–10 min [34,35].

Al-Maswary et al. [36] describe the effects of ultrasound, such as progenitor proliferation (Ki67+, β-tubulin+, neurofilament), ERK activation, neuronal differentiation, and cognitive improvement. The parameters provided are 1.5 MHz, 60 mW/cm^2^ (I_SATA), PRF 100 Hz, duty cycle 20%, exposure 20 min (single or 3×).

The results indicate that exposure to LIPU induced an increase in the number of NSCs. The ERK signaling pathway is related in the process of neural stem cell proliferation induced by LIPU, as evidenced by the simultaneous inhibition of phosphorylated ERK levels and cell proliferation in the attendance of a MEK/ERK pathway inhibitor.

The results of LIPUS stimulation [37] showed that a 69.3 mW/cm^2^ intensity (1 MHz, 8 V) was sufficient to promote the expression of growth factors in neural stem cells (NSCs) in vitro. LIPUS not only reduced differentiation of astrocyte but also stimulated their differentiation into neurons. Furthermore, LIPUS stimulation significantly increased the expression levels of Notch1, Hes1, and BDNF. The results of this study suggest that LIPUS stimulates the proliferation and differentiation of neural stem cells by modulating the Notch signaling pathway. LIPUS may be a potential therapeutic platform for optimizing the conditions for stem cell proliferation and maturation and enabling non-invasive neuromodulation in diseases of the central nervous system.

Another study demonstrating the effect of ultrasound on neurogenesis is the work by Ye et al. [38]. The study investigated the effect of various LIPUS parameters—namely, duty cycle, spatially and temporally averaged intensity (SATA), and ultrasound amplitude—on the therapeutic effectiveness of SK-N-SH cells cultured under serum-deprived conditions. Four different LIPUS settings were used: 50 mW/cm^2^ (A), 40%, 25 mW/cm^2^ (B), 10%, 50 mW/cm^2^ (C), 20%, and 25 mW/cm^2^ (D), 10%. Analysis revealed promotion of neurite outgrowth in all LIPU-treated groups, with the exception of group D. In addition, it was found that LIPU treatment effectively stimulates the expression of BDNF and increases the phosphorylation of protein kinase B (Akt), the extracellular signal-regulated kinase (ERK) 1/2, and mTOR signaling pathways. It is worth noting that these same factors and pathways influence neurogenesis, indicating that they occur simultaneously in the process of neuroplasticity.

Table 1 presents the mechanisms by which ultrasound affects neurogenesis.

### 2.5. Intermittent Hypoxia (IHT)

Research on hypoxia-induced neuroprotection has demonstrated the potential to accelerate post-stroke recovery through adaptive cellular responses. This process is enhanced by techniques involving passive, intermittent exposure to hypoxia (Intermittent Hypoxic Exposure—IHE) or intermittent exposure to hypoxia followed by hyperoxia (Intermittent Hypoxic–Hyperoxic Exposure—IHHE), with alternating low and normal/high oxygen concentrations [39,40].

The cited review [39] most important information and key areas in which IHE/IHHE exhibits adaptive responses, including hypoxia-inducible factor (HIF) signaling, regulation of reactive oxygen species (ROS), and mitochondrial energetics, contributing to improved neuroprotection and regeneration of the tissue. IHE/IHHE improves cerebral blood flow and promotes angiogenesis, facilitating vascular remodeling and enhancing perfusion in damaged brain areas. IHE/IHHE enhances neurogenesis and functional regeneration, supporting brain repair by promoting neuronal longevity and regeneration. IHE/IHHE has also been shown to improve cognitive and motor function in post-stroke patients, as well as in older adults with mild cognitive impairment. IHE/IHHE decreases cardiovascular risk factors. Further research is needed to optimize the IHHE protocols and determine their long-term effects, and effectively include them into clinical practice, particularly in the context of neurogenesis and neuroplasticity.

An endogenous mechanism, which is hypoxic preconditioning (HPC), has been used to increase the expression of BDNF, a marker of depression, to induce a neuroprotective effect in the treatment of mood disorders [40].

### 2.6. The Effect of Hyperbaric Oxygen Therapy on Neurogenesis

Numerous studies indicate that hyperbaric oxygen therapy (HBOT) promotes neurogenesis. Hyperbaric oxygen therapy includes inhaling 100% O_2_ at a pressure of 1.5–3.0 ATA for 60–120 min, 5 times a week, for 20–40 sessions. Effects are visible after 10–20 sessions, with maximum effects observed after 40 sessions. HBOT stimulates neurogenesis in the adult brain, particularly in SVZ) and the hippocampus in DG (as well as in the cerebral cortex following injury) [41].

In vitro studies indicate that hyperbaric oxygen therapy (HBOT) promotes the differentiation of neural stem cells into oligodendrocytes or neurons, while inhibiting their differentiation into astrocytes. HBOT also increases the proliferation of other supporting cells, including cells expressing glial cell line-derived neurotrophic factor (GDNF) and cells expressing vascular endothelial growth factor (VEGF), and also epithelial cells and human microvascular endothelial cells (HMEC-1).

Documented effects (including clinical trials confirmed by a review of numerous publications) involve the proliferation of progenitor cells (BrdU+, DCX+, Sox2+), differentiation into mature neurons (NeuN+, MAP2+), increased survival of new neurons, and increased hippocampal volume (MRI in humans) It improves memory, executive functions, and mood. Importantly, short-term hyperoxia is pro-neurogenic, whereas prolonged exposure may be toxic due to an increase in ROS (reactive oxygen species), whose excess leads to oxidative stress, damaging DNA, proteins, and lipids; therefore, intermittent sessions are recommended [41].

Early treatment with hyperbaric oxygen may reduce delayed memory impairments following acute carbon monoxide poisoning by maintenance neurogenesis in adults through an increase in levels BDNF in the hippocampus [42].

HBOT affects numerous cellular pathways and functions, including mitochondrial biogenesis and function (increased Bcl-2 production, decreased Bax production, and increased ATP production), neurogenesis (increased Wnt-3 expression and VEGF/ERK signaling), synaptogenesis (increased expression of GAP43 and synaptophysin), and anti-inflammatory responses (decreased expression of TNF-α and IL-6). The VEGF/ERK signaling pathways are key, interrelated molecular pathways that dynamically regulate neurogenesis in adult mammals. The above markers control the balance between maintaining the viability of neural stem cells (NSCs), stimulating their division (proliferation), and directing them toward maturation into mature neurons (differentiation). These mechanisms lead to significant clinical benefits, such as improved cognitive function, enhanced post-concussion syndrome, recovery from traumatic brain injury, and reduced symptoms in terms such as post-traumatic stress disorder and fibromyalgia [43].

The effect of HBOT can be explained by the fact that neurogenesis is an energy-intensive process; therefore, blood vessels are an important part of the neurogenic niche, as they provide the necessary oxygenation of progenitor cells [44].

### 2.7. The Effect of Whole-Body Cryotherapy (Cryochambers) on Neurogenesis

Therapeutic hypothermia (TH) is currently the standard of care for hypoxic–ischemic encephalopathy (HIE) in developing countries; nevertheless, it is not always effective, particularly in severe cases of HIE. Approximately 80% of studies have shown significant improvement in cognitive and/or sensorimotor functions, as well as a reduction in brain damage, following stem cell transplantation (SCT) in the treatment of HIE in combination with TH [45].

A study [46] observed a significant increase in serum irisin levels as early as one hour after the first WBC (Whole Body Cryotherapy) session, especially among younger participants. This effect persisted throughout the study protocol. Irisin concentrations increased in response to chronic WBC, while BDNF levels were left unchanged, which is somewhat inconsistent with previous reports. The study does not confirm an indirect effect of WBC on neurogenesis.

Between groups were found significant differences in terms of cognitive function, favoring the WBC group. An improvement in mood was noted based on self-assessment of depression symptom severity. A significant decrease in BDNF levels was observed (*p* < 0.05). WBC may improve cognitive function. In future studies on early intervention methods for mild cognitive impairment (MCI), combining WBC with existing behavioral and cognitive training appears promising [47].

A study [46] observed a significant increase in serum irisin levels as early as one hour after the first WBC session. Neurogenesis (the formation of new neurons, most importantly in the adult hippocampus) is difficult to measure directly in living humans. The literature on PubMed regarding WBC and neurogenesis is very limited—there are no studies that directly assess neurogenesis (e.g., using specific biomarkers such as double-cortin, or imaging markers of new neuron formation). The evidence is entirely indirect and focuses on cognitive outcomes in mild cognitive impairment (MCI), effects on mood, or circulating neurotrophic/neuroplasticity markers, such as BDNF, which supports neuronal survival, synaptic plasticity, and (indirectly) neurogenesis [48].

### 2.8. The Effect of Light Therapy on Neurogenesis

Near-infrared (NIR) light effectively promoted the proliferation of neural stem cells (NSCs) and the differentiation of astrocytes via the OPN4 photoreceptor. Furthermore, NIR stimulation significantly enhanced neurogenesis and calcium-dependent astrocyte activity. Moreover, activation of hippocampal astrocytes with near-infrared (NIR) light at a frequency of 40 Hz significantly improved anxiety-related behaviors in mice.

It was found that pulsed near-infrared (NIR) light (940 nm/40 Hz) enhances neurogenesis in the hippocampus, exerting an anxiolytic effect. This innovative approach shows promise as a new preventive treatment for depression [48].

Photobiomodulation (PBM) is a non-invasive technique that uses light to modulate cellular processes and promote tissue regeneration. As a regenerative therapy, PBM has emerged as a promising approach for promoting the differentiation of adipose-derived stem cells into neurospheres, which are a round cluster of neural stem and progenitor cells. PBM influenced the growth of neurospheres, mitochondrial function, and cell differentiation, with particular emphasis on wavelengths of 825 and 525 nm and fluences of 5 and 10 J/cm^2^. PBM modulates the size of neurospheres and growth kinetics, while different effects were observed with varying combinations of wavelengths and fluences. The 825 nm wavelength at 5 J/cm^2^ promoted the formation of larger neurospheres with a slower growth rate, whereas 525 nm at 10 J/cm^2^ induced the formation of smaller, rapidly differentiating clusters. A wavelength-dependent effect on mitochondrial function and cellular differentiation was observed, accompanied by increased expression of the β-tubulin III and NeuN genes up to day 11. The effect of PBM on cell behavior is multifaceted, promoting differentiation and modulation of mitochondrial function. PBM has potential as a non-invasive approach in stem cell-based therapies aimed at neuronal repair and regeneration and warrants further investigation into its mechanisms and applications [49].

Brain photobiomodulation (PBM) therapy using red and near-infrared (NIR) light is an innovative method for treating a wide range of neurological and psychological conditions. Red/NIR light stimulates complex IV of the mitochondrial respiratory chain (cytochrome c oxidase) and increases ATP synthesis. Furthermore, light absorption by ion channels triggers the release of Ca^2+^ ions, leading to the activation of transcription factors and gene expression. PBM therapy of the brain increases the metabolic efficiency of neurons and stimulates anti-inflammatory, anti-apoptotic, and antioxidant responses, as well as neurogenesis and synaptogenesis. Its therapeutic role in disorders such as dementia and Parkinson’s disease, as well as in the treatment of stroke, brain injury, and depression, is gaining increasing attention. In transcranial PBM therapy, delivering a sufficient dose to achieve optimal stimulation is difficult due to the exponential attenuation of light penetration into the tissue. Alternative approaches, such as intracranial and intranasal light delivery methods, have been proposed to overcome this limitation. This article reviews the latest preclinical and clinical data on the efficacy of brain PBM therapy. Brain PBM increases neuronal metabolism, stimulates neurogenesis and synaptogenesis via CCO → ATP + BDNF + reduction in inflammation [50].

### 2.9. Massage/Mechanical Vibration

Massage and neurogenesis are a topic that has been the subject of far fewer direct studies than, for example, photobiomodulation or physical exercise. Most of the scientific literature does not show that massage directly induces neurogenesis in healthy adults, but points to indirect mechanisms that support neuroplasticity, the growth of neurotrophic factors (such as BDNF, IGF-1), and improvement of the brain environment (reduction in stress and inflammation, better regulation of the HPA axis) [51]. Given the potential ability of flavonoids to chelate divalent cations [52]. It is also worth highlighting their role in the onset and progression of neurodegenerative diseases [53]. This is significant for the further advancement of knowledge and potential investment in this field.

Traditional pediatric massage (TPM) exerts a marked antidepressant effect in adolescent rats subjected to CUMS by inhibiting excessive HPA axis activity (decreased CORT), enhancing the expression of glucocorticoid receptors (GR) and the neurotrophic factors such as BDNF and IGF-1 in the hippocampus [54].

The relationship between massage and neurogenesis (the formation of new neurons, primarily in the hippocampus) is a topic that has been the subject of significantly fewer direct studies than, for example, photobiomodulation or physical exercise. Most of the scientific literature does not show that massage directly induces neurogenesis in healthy adults, but points to indirect mechanisms supporting neuroplasticity, the growth of neurotrophic factors (such as BDNF, IGF-1), and improvement of the brain environment (reduction in stress and inflammation, better regulation of the HPA axis) [51].

Table 2 presents physical factors that stimulate neurogenesis.

## 3. Discussion

Neurogenesis, as a neurobiological process, is in itself an extraordinary phenomenon, demonstrating the ability of nerve tissue to regenerate and proliferate—something long considered impossible. Even more remarkable is the fact that it is part of a highly complex process capable of occurring in the brains of animals and humans, serving the broader purposes of compensation and adaptation to changing conditions both within and outside the brain.

Neuroplasticity, broadly classified as structural, functional, biochemical, and behavioral, encompasses synaptogenesis and neurogenesis. Functional neuroplasticity operates through LTP and long-term depression (LTD). Biochemical neuroplasticity includes genes such as apoE and BDNF. Behavioral neuroplasticity involves the prefrontal cortex and the nucleus accumbens—two brain regions that contribute to the beneficial effects of certain psychotropic drugs by exhibiting plastic changes [30]. Mirror neurons play a key role in neurorehabilitation by utilizing the process of functional synaptic plasticity. Non-invasive brain stimulation, including repetitive transcranial magnetic stimulation (rTMS) [26], deep brain stimulation [27], tDCS [28], and scrambler therapy [56] for pain management, utilizes neuroplasticity in the treatment of numerous disorders, such as stroke, autism, Parkinson’s disease, and depression.

BDNF is released in response to most physical stimuli. Among the neurotrophins, including nerve growth factor (NGF), neurotrophin-3 (NT-3), and neurotrophin-4 (NT-4/5), BDNF is the subject of extensive research regarding its role in cell survival as well as synaptic control in neurons of the CNS. BDNF binds to a tyrosine kinase (TrkB), and the intracellular signaling cascades resulting from this binding play a crucial role in determining cellular fate, including the differentiation and maturation of CNS neurons [57].

It has been shown that reduced expression or dysregulation of the BDNF/TrkB system is associated with the pathogenesis of neurological and psychiatric disorders, such as depression and ALZ. Interestingly, the effects of BDNF-mimetic compounds, including flavonoids —small molecules that can activate TrkB-mediated signaling—has been extensively studied as a possible therapeutic strategy for brain diseases, given that the neurotrophin receptor p75 (p75NTR), a common neurotrophin receptor, also take part to cell death in various pathological conditions, such as neurodegeneration. Since reduced activity of the BDNF/TrkB system relates to the pathophysiology of psychiatric disorders and neurodegenerative diseases, understanding how changes in the BDNF/TrkB system contribute to disease progression may provide valuable insights regarding the prevention of these diseases. on the potential impact of BDNF mimetics on the pathophysiology of brain diseases [57]. BDNF is difficult to use therapeutically (short half-life, poor penetration of the blood–brain barrier), which is why mimetics that activate the TrkB receptor (or sometimes p75NTR) are being developed [58].

As mentioned, neurotrophic factors influence not only neurogenesis but also axonal regeneration. In a study by Neuman K et al. [55], the effect of direct current magnetic stimulation on the initial growth of neurons in vitro was quantified. After 3 days of 1-h magnetic field stimulation, a 62.5% increase in the area of neurite outgrowth was observed compared to unstimulated controls, and this resulted in a directional deviation of growth parallel to the direction of the magnetic field. At the same time, varying magnetic field conditions did not cause changes in two neurotrophic factors: NGF and BDNF, released from glial cells. These results indicate the potential of direct current magnetostimulation in stimulating neuronal regrowth and improving clinical outcomes. The results obtained suggest that the regenerative effect of magnetic stimulation is not solely due to the stimulation of neurotrophic factors but is related to the conditions under which the magnetic field is applied. Under the parameters described, regeneration occurred without changes in BDNF and NGF [55].

The hippocampus plays a role in the etiopathogenesis of depression and in the antidepressant effects of medications. Hippocampal plasticity manifests as neurogenesis in adults and is responsible for depression-related behaviors in animal models. Chronic application of electroconvulsive therapy (ECT) strongly promotes neurogenesis; however, less is known about the neurogenic symptoms of other forms of stimulation therapy, such as repetitive rTMS. This study examined the time course of the acute effects of ECS (less frequently studied than chronic effects) and rTMS on markers of cell proliferation and neurogenesis in the adult hippocampus. Mice underwent a single session of 10 Hz rTMS or theta-burst stimulation (iTBS). Following ECS stimulation, the mice exhibited a transient increase in cell proliferation after 3 days. Seven days after stimulation, an increase in the number of proliferating neuronal precursor cells was observed. The results suggest that neurostimulation therapies have different effects on hippocampal neurogenesis, where ECS may have various effects on neurogenesis in the hippocampus, where the endocannabinoid system (ECS) may exert greater neurogenic effect than 10-Hz iTBS and rTMS. A single stimulation session may be insufficient to achieve a long-lasting therapeutic effect [59].

Biomarkers are intended to be used to track various pathophysiological processes in OA, such as angiogenesis, neurogenesis, inflammation as well as cartilage degeneration. Several serum markers are already elevated in the early stages of OA, when X-rays show no or minimal structural changes in the knee joint. Vascular VEGF, a mediator that could may be used as a biomarker promoting angiogenesis, is correlated with the severity of OA. Interestingly, NGF is also elevated in the subchondral bone of patients with OA and has therefore been identified as a specific target for OA treatment. Since patellofemoral pain (PFP) is considered a precursor to OA, and changes in inflammatory, angiogenic, and neurogenic biomarkers are already present in PFP, just as in early OA. NGF was elevated in PFP patients with greater pain caused by abnormal patellofemoral joint alignment. It is worth noting the presence of this mediator in peripheral tissues in symptoms not resulting from CNS damage [60].

Physical methods are increasingly being used in therapy thanks to our understanding of their effects. NSCs could proliferate, self-renew, and differentiate in multiple directions. However, they do not always survive or differentiate into mature neurons capable of replacing lost neurons. In a study by Liao YH et al. [61], the potential role of LIPU in promoting the proliferation and differentiation of NSCs, as well as its role in the healing of spinal cord injuries (SCI), was evaluated.

LIPU significantly increased the expression of key neurogenic markers and neurotrophic factors, NSC proliferation, while reducing glial fibrillary acidic protein (GFAP) expression and preventing apoptosis. In vivo, the NSC/LIPU+ group demonstrated higher chances of survival and differentiation of transplanted NSCs, along with improved BBB outcomes and increased expression of neuronal markers compared to the NSC/LIPU- group. LIPU stimulation effectively promoted the proliferation and differentiation of NSCs as well as increased the survival and function of transplanted NSCs at the site of spinal cord injury, leading to a faster recovery from spinal cord injury [61].

While the proliferation and differentiation of NSCs are generally undisputed, the question of whether stem cells or neurons migrate remains a subject of research. Evidence supporting the migration of stem cells is found in the study by Cicchetti et al. [62], carried out using magnetic resonance imaging (MRI) and positron emission tomography (PET). After 3 weeks, minimal migration of cells implanted into the striatum was observed, whereas SVZ cells implanted into the RMS migrated toward the olfactory bulb as early as 1 week after transplantation. Live SPIO-labeled neuronal cells were observed 7 weeks after transplantation. Using double immunostaining differentiation into neuronal and astrocytic phenotypes was demonstrated. The phenomena described indicate the potential for migration and differentiation of stem cells [62].

The limitations of this study stem from the likelihood that the most relevant research findings may not have been cited, as both the volume of such findings and the pace of progress in this field are currently very high. Similarly, it was not possible to explain the mechanisms involved in neurogenesis or to provide detailed context for the research, as this would have significantly increased the scope of the work. Therefore, the study primarily presents research findings and conclusions. It is also difficult to standardize the various results due to the application of physical factors in different doses. The nature of the analysis calls for systematic reviews on this topic, as prevention and treatment with these factors are safe, inexpensive, and promise good results.

## 4. Conclusions and Future Research

As our understanding of the properties of physical factors continues to grow, we are better able to harness environmental factors to regulate neurogenesis in both health and disease. Some of the factors studied, when appropriately selected, can be effectively used in the treatment of neurodegenerative diseases, central nervous system injuries, and in adaptation to specific conditions, or to improve cognitive processes in relation to the regulation of neurogenesis. On the other hand, a good understanding of physical conditions can protect against the adverse effects of certain phenomena or some parameters of them on the conditions for the proliferation and maturation of nerve cells, a point that has not yet received sufficient attention.

## Figures and Tables

**Table 1:** Mechanisms by which ultrasound affects neurogenesis [20,34,35,36,37,38].

Mechanisms	Description	Key Mediators	Type of Study
Mechanical effect (acoustic wave radiation)	Pressure waves that induce microcurrents in cell membranes → opening of ion channels	↑ Intracellular Ca^2+^	Preclinical/mechanistic studies, in vitro and animal studies
Stable cavitation	Oscillating microbubbles → local shear forces → activation of mechanoreceptors	Integrins, PIEZO1	Preclinical/mechanistic studies, in vitro and animal studies
Activation of signaling pathways	Expression of BDNF, VEGF, and NGF → promotes the survival and differentiation of NSCs	ERK1/2, Akt, CREB, mTOR	Studied indirectly/preclinically or in animals
Modulation of the blood–brain barrier (BBB)	Temporary opening of the BBB → influx of trophic factors and progenitor cells	↑ Permeability of tight junctions	Human studies
Reduction in inflammation	↓ pro-inflammatory microglia (M1) → ↑ neuroprotective microglia (M2)	↓ TNF-α, IL-1β, ↑ IL-10	Human studies, partial/indirect
Mitochondrial stimulation	↑ mitochondrial biogenesis → more ATP for cell proliferation	PGC-1α	In vitro studies on human astrocytes and animal models

Note: If the intensity is too high (>1 W/cm^2^), thermal damage or inertial cavitation may occur → inhibiting neurogenesis.

**Table 2:** Physical factors that stimulate neurogenesis.

L.P.	Factor	Mechanism of Action	References
1	Physical exercise (aerobic, strength training)	↑ BDNF, IGF-1, VEGF ↑ Myokines (irisin) ↑ Gpld1 ↓ Cortisol ↑ Blood flow	[22,23,24] Human studies Neurogenesis is inferred indirectly (from biomarkers and imaging)
2	Magnetic fields (TMS, tDCS, PEMF)	↑↓ Modulation of neuronal excitability ↑ Development and maturation of newly formed neurons ↑ BDNF ↑ NGF ↑↓ Modulation of glutamate and GABA ↑ 5-HT, DA, NA, ACh	[25,26,27] Human studies
3	Electrostimulation (tDCS, tES, tACS, tRCS)	↑↓ Modulation of neuronal excitability ↓↑ NMDA ↓↑ GABA No changes in BDNF, NGF	[28,30,31,32,55] Human studies
4	Low-intensity focused ultrasound (LIFUS)	↑ Intracellular Ca^2+^ ↑ BDNF, VEGF, NGF, IL-10 ↓ TNF-α, IL-1β ↑ Proliferation of progenitor cells (BrdU+, Nestin+), ↑ Differentiation into neurons (β-III-tubulin+, NeuN+) ↑ Migration of new neurons to the cortex and hippocampus Additional data in Table 1	[20,34,35,36,37,38] Human studies Preclinical/mechanistic studies, in vitro and animal studies
5	Intermittent hypoxia	↑↓ IHE/IHHE influences adaptive responses, including HIF signaling, ROS regulation, and mitochondrial energy metabolism. ↑ Angiogenesis and vascular remodeling ↑ VEGF Cerebral blood flow, ↑ Vascular remodeling ↑ Perfusion in damaged brain areas ↑ BDNF Neurogenesis, brain repair, and functional regeneration ↑ Neuron survival and regeneration ↑ Cognitive and motor functions	[39,40] Human studies Animal studies
6	HBOT, Hyperbaric Oxygen Therapy	↑ Neurogenesis—by increasing oxygen availability in tissues, which activates numerous molecular and cellular pathways ↑ VEGF, ERK ↑ BDNF, GDNF, HIF-1α (Hypoxia-Inducible Factor-1α), activation of HIF-1α and β-catenin, although excess may be toxic ↓ Inflammatory factors (e.g., IL-10, TNF-α)—HBOT reduces inflammation	[41,42,43,44] Human studies Animal studies
7	Cryotherapy (WBC whole-body cryotherapy, cryostimulation)	↑ BDNF, irisin, endorphins, norepinephrine; ↓ IL-6, TNF-α; adaptive response	[45,46,47,48] Human studies Animal studies
8	Light therapy (phototherapy) PBM photobiomodulation at 825 and 525 nm, 5 and 10 J/cm^2^. NIR—near-infrared 940 nm/40 Hz	↑ NSC proliferation and astrocyte differentiation → OPN4 photoreceptor ↑ Expression of β-tubulin III and NeuN genes ↑ Neuronal metabolism ↑ Neurogenesis and synaptogenesis via CCO → ATP + BDNF ↓ Reduction in inflammation ↑ Melatonin ↑ BDNF	[48,49,50] Human studies Animal studies
9	Massage/mechanical vibrations	Proprioceptive stimulation → ↑ BDNF, IGF ↓ CORT	[51,54] Human studies Animal studies

## Data Availability

No new data were created or analyzed in this study. Data sharing is not applicable to this article.
